# Skin-Colored Papules on the Neck of a Postmenopausal Woman: A Diagnostic Challenge

**DOI:** 10.3390/dermatopathology12020015

**Published:** 2025-05-14

**Authors:** Jason El Jalkh, Pia Maria Obeid, Dorra Guermazi, Aya Soubra, Elie Saliba

**Affiliations:** 1 Department of Dermatology, Gilbert and Rose-Marie Chagoury School of Medicine, Lebanese American University, Beirut 13-5053, Lebanon; jason.eljalkh01@lau.edu (J.E.J.); piamaria.obeid@lau.edu (P.M.O.); 2 Department of Dermatology, The Warren Alpert Medical School of Brown University, Providence, RI 02903, USA; dorra_guermazi@brown.edu; 3 Department of Pathology, Gilbert and Rose-Marie Chagoury School of Medicine, Lebanese American University, Beirut 13-5053, Lebanon; aya.soubra@laumcrh.com

**Keywords:** pseudoxanthoma elasticum-like papillary dermal elastolysis, acquired elastic tissue disorder, trifarotene therapy, histopathology, dermatopathology

## Abstract

A 64-year-old patient presented for management of symptomatic skin-colored papules symmetrically distributed over the lateral neck over the past two years, which failed to improve on multiple topical corticosteroids, antifungal creams, and topical calcineurin inhibitor. Histopathologic examination showed a regular epidermis with increased melanophages in the papillary dermis, without vacuolar degeneration of the basement membrane. Verhoeff Van Gieson stain highlighted a band-like zone of attenuated elastic fibers in the papillary dermis, while Von Kossa stain was negative for calcified fibers. PAS staining was negative for fungal organisms and Alcian blue showed no increase in dermal mucin.

## 1. Case Presentation

**Figure 1:**
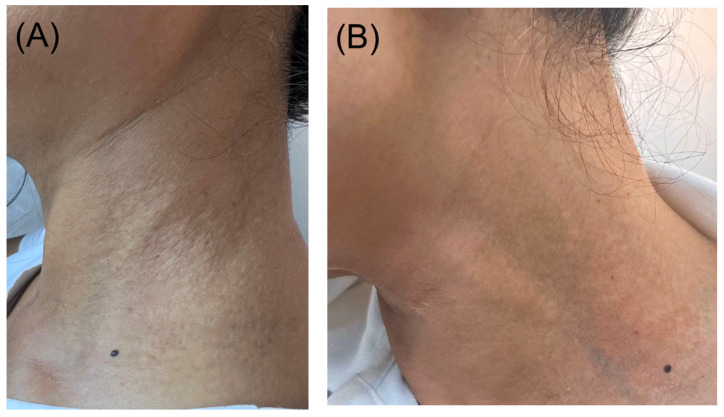
(**A**) Dome-shaped skin-colored papules and plaques over the lateral neck. (**B**) Marked improvement after treatment.

A 64-year-old patient with a medical history of irritable bowel syndrome, depression, and endometriosis presented for management of symptomatic lesions on her neck, which has been present for the past 2 years. The patient denied significant sun exposure in the past years but reported that heat exposure triggered itching and burning sensation in the affected area. On physical examination, skin-colored papules were noted, symmetrically distributed over the lateral neck (Figure 1A).

Over the past two years, the patient has attempted various treatments, including multiple topical corticosteroids, antifungal creams, and topical calcineurin inhibitor, without achieving symptomatic relief or clinical improvement in the lesions.

**Figure 2:**
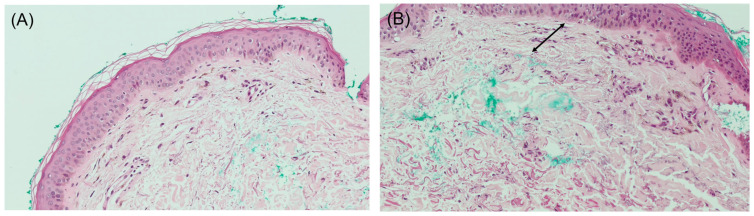
(**A**) Biopsy shows a regular orthokeratotic epidermis with increased dermal melanophages (H&E, 10×). (**B**) High-power magnification showing attenuated elastic fibers in the papillary dermis (arrow) (H&E, 20×).

**Figure 3:**
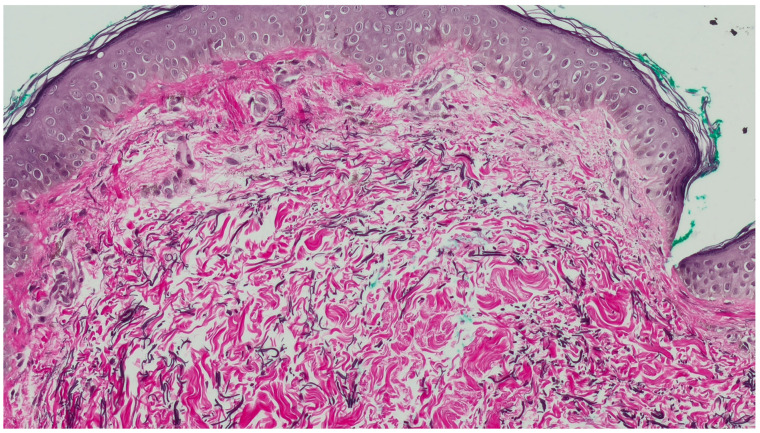
Verhoeff Stain (elastic stain) highlights the curved elastic fibers of the reticular dermis. Note the attenuation of the elastic fibers in the papillary dermis (Elastic stain, 20×).

**Figure 4:**
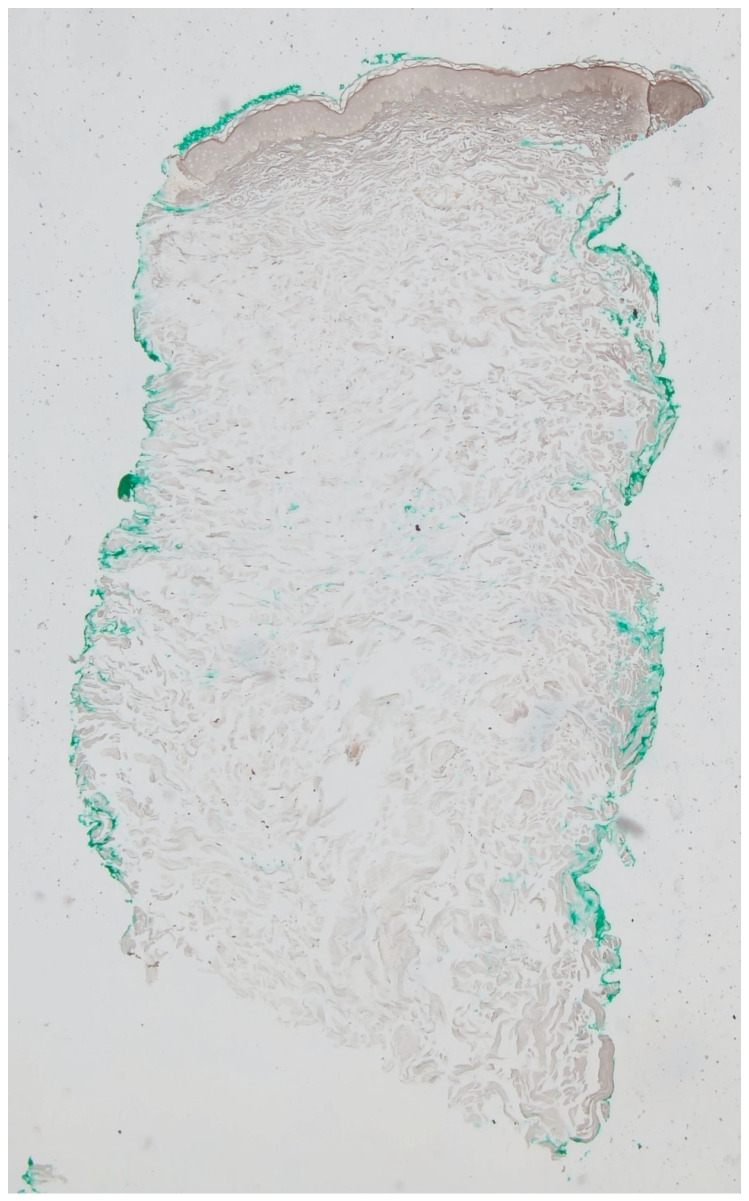
Von Kossa staining of the tissue sample, highlighting the absence of calcium deposits at the level of the mid-dermis (Von Kossa, 5×).

A 3 mm punch biopsy was performed on the right lateral neck. Histopathologic examination showed a regular epidermis with increased melanophages in the papillary dermis, without vacuolar degeneration of the basement membrane (Figure 2A,B). Given that the findings on hematoxylin–eosin (H&E) largely resembled normal skin and the clinical presentation mimicked PXE, additional stains were performed to aid in diagnosis. Verhoeff Van Gieson stain highlighted a band-like zone of attenuated elastic fibers in the papillary dermis (Figure 3), while Von Kossa stain was negative for calcified fibers (Figure 4). PAS staining was negative for fungal organisms and Alcian blue showed no increase in dermal mucin.

## 2. What Is the Diagnosis?

 **A.** Pseudoxanthoma elasticum;
 **B.** Pseudoxanthoma elasticum-like papillary dermal elastolysis;
 **C.** White fibrous papulosis;
 **D.** Papillary dermal elastosis;
 **E.** Mid-dermal elastolysis.

## 3. Diagnosis

### Pseudoxanthoma Elasticum-like Papillary Dermal Elastolysis

The patient was advised to apply trifarotene cream, one pump daily on each side of the neck, alternating with a topical corticosteroid cream to minimize irritation. Three months later, she reported improvement in both pruritus and clinical lesions (Figure 1B).

## 4. Discussion

Our article contributes a new case of PXE-PDE to the literature. This entity was first described in 1992 by Rongioletti and Rebora [1]. Clinically, it is characterized by non-follicular, dome-shaped, soft, yellowish papules measuring 2 to 3 mm, which coalesce into plaques and show predilection for the lateral and posterior neck areas [2]. In the most recent case series, 100% of reported PXE-like PDE cases were asymptomatic, unlike our patient, who experienced pruritus since lesion onset. While PXE-like PDE is typically asymptomatic, symptomatic cases have been documented, including a 65-year-old Caucasian woman who reported mild itching since lesion development in 2013 [1].

Since its initial description, few cases of PXE-like papillary dermal elastolysis (PDE) have been reported [3]. Although a case has been described in a middle-aged woman, most cases affect postmenopausal women, consistent with our case. Notably, no cases in the literature have involved men [4,5].

The pathogenesis of PXE-like PDE is thought to involve abnormal elastic fiber formation, intrinsic aging, and ultraviolet (UV) radiation [6,7]. UV radiation is a contributing factor in PXE-like PDE, though it does not fully explain the disease’s pathogenesis, as over 30% of cases affect non-sun-exposed areas like the axilla, and more than half of patients report no history of prolonged sun exposure [4]. Histologic evidence supports UV radiation’s role, with increased melanophages in sun-exposed skin compared to protected areas [8], and melanophages in the papillary dermis in nearly all reported cases [4]. This suggests that repeated UV-induced injury at the dermo-epidermal junction, as seen in chronic heliodermatitis, could drive pathologic changes [9]. However, our patient did not report significant sun exposure, reflected by the lack of solar elastosis on pathology.

Intrinsic aging is another factor, with loss of both elastin and fibrillin-1 in the dermis [3]. Fibrillin-1 is also absent in the neck skin of elderly individuals, potentially contributing to dermal component loss in PXE-like PDE [7]. In the previous literature, approximately 40% of PXE-like PDE cases have not been linked to systemic conditions. In the most recent case series, the distribution of systemic diseases was as follows: 17.6% with hypertension, 11.8% with hyperlipidemia, and 5.9% each with hypothyroidism, anemia, and atopic disease [4]. However, these comorbidities appear to be related to natural aging in postmenopausal women and not directly linked to PXE-PDE. Notably, our patient is a 64-year-old postmenopausal woman with no significant systemic comorbidities.

Histologically, hematoxylin–eosin staining often reveals minimal findings, with the skin appearing nearly normal (invisible dermatosis). Thus, in the absence of clear clinicopathologic correlation, diagnosis can be easily missed. Special stains for elastic fibers such as orcein, Verhoeff Van Gieson, or Weigert are essential for confirmation. The epidermis is typically normal or slightly thinned, with the most characteristic finding being a band-like loss or marked reduction in eulanin and oxytalan elastic fibers in the papillary dermis, often accompanied by sparse melanophages within the zone of elastic loss. Additionally, a mild perivascular lymphocytic infiltrate in the papillary dermis may be observed [4].

The presence of melanophages without vacuolar degeneration in this case likely reflects a secondary response to chronic epidermal stress, mechanical friction, and age-related dermal changes, in addition to the PXE-like pathology.

The primary elastic tissue disorders in the clinical and histologic differential diagnosis of PXE-like papillary dermal elastolysis (PXE-PDE) include pseudoxanthoma elasticum (PXE), white fibrous papulosis, papillary dermal elastosis, and mid-dermal elastolysis. PXE presents clinically similarly to PXE-PDE but is distinguished by associated comorbidities. Histologically, elastic changes are found in the reticular dermis with sparing of the papillary dermis, showing fragmented, calcified elastic fibers without melanophages [4]. White fibrous papulosis is characterized by non-confluent, whitish, firm papules on the posterior neck. Histologic findings may include loss of elastic fibers with dermal fibrosis and possible melanophages [10,11]. Papillary dermal elastosis has a similar clinical appearance to PXE-PDE. Histologically, it shows focal clumps of elastic fibers alternating with areas of oxytalan and elaunin fiber loss, along with an increase in elastic fibers in the reticular dermis. Melanophages are typically absent [12]. Finally, mid-dermal elastolysis presents with well-circumscribed patches of fine wrinkles or perifollicular papules on the trunk and proximal extremities. Histologic findings are confined to the reticular dermis, with a loss of elastic fibers and no melanophages present [13].

## 5. Conclusions

PXE-like PDE is a chronic, slowly progressing condition with limited effective treatments. Therapeutic attempts with topical retinoids have generally shown minimal improvement, although one isolated report indicated marked improvement. Intralesional triamcinolone provided no clinical benefit in one case, despite two rounds of treatment. Lasers, particularly ablative CO_2_ lasers, have shown promise by improving skin texture and appearance by approximately 50%, as reported by a patient in 2018 [14]. Given the lack of established treatments for PXE-like conditions, the use of this retinoid is exploratory. The mix between a retinoid and a steroid was to mitigate potential irritation given the sensitivity of the neck area where the lesions appeared. Notably, our patient achieved significant improvement with trifarotene cream, introducing a new therapeutic option for PXE-like PDE not previously documented in the literature.

## Data Availability

The original contributions presented in this study are included in the article. Further inquiries can be directed to the corresponding author.
